# From agent-based models to the macroscopic description of fake-news spread: the role of competence in data-driven applications

**DOI:** 10.1007/s42985-022-00194-z

**Published:** 2022-10-03

**Authors:** J. Franceschi, L. Pareschi, M. Zanella

**Affiliations:** 1 Department of Mathematics “F. Casorati”, University of Pavia, Pavia, Italy; 2 Department of Mathematics and Computer Science, University of Ferrara, Ferrara, Italy

**Keywords:** Fake news spreading, Learning dynamics, Agent-based models, Kinetic models, Social closure, Competence, Data uncertainty, 35Q91, 65C05, 82C40, 91D30

## Abstract

Fake news spreading, with the aim of manipulating individuals’ perceptions of facts, is now recognized as a major problem in many democratic societies. Yet, to date, little has been understood about how fake news spreads on social networks, what the influence of the education level of individuals is, when fake news is effective in influencing public opinion, and what interventions might be successful in mitigating their effect. In this paper, starting from the recently introduced kinetic multi-agent model with competence by the first two authors, we propose to derive reduced-order models through the notion of social closure in the mean-field approximation that has its roots in the classical hydrodynamic closure of kinetic theory. This approach allows to obtain simplified models in which the competence and learning of the agents maintain their role in the dynamics and, at the same time, the structure of such models is more suitable to be interfaced with data-driven applications. Examples of different Twitter-based test cases are described and discussed.

## Introduction

Since the 2016 U.S. presidential election, and more recently the COVID-19 infodemic, fake news on social networks, intended to manipulate users’ perceptions of events, has been recognized as a fundamental problem in open societies. As fake news proliferate, disinformation threatens democracy and efficient governance. In particular, there is empirical evidence that fake news spreads significantly “ faster, deeper, and more widely” than real news [37]. In the same study, it is also highlighted that the phenomenon is not due to robotic automatisms of news dissemination but to the actions of human beings sharing the news without the ability to identify misinformation.

It is therefore of fundamental importance the construction of mathematical models capable of describing such scenarios and with a structure simple enough to be interfaced with data available, for example from social networks, but still embedding the specific features related to the ability of individuals in detecting the piece of false information.

In recent years, compartmental models inspired by epidemiology have been used fruitfully to study spreading phenomena of rumors and hoaxes. For instance, following the pioneering work of Daley and Kendall [11], in [23] SIR-type models are used in conjunction with dynamical trust rates that account for the different spreading rates in a network. Those traditional models were elaborated in [7], where the authors consider also the impact of online groups in feeding the rumor growth once it has started.

Alongside these approaches there are more data-driven works. In this field, Twitter has been gaining consensus as a powerful source of useful and structured information. A recent example in this direction can be found in [27], that focuses on fake news dissemination on the platform using a two-phase model, where fake news initially spread as novel news story and after a correction time they are paired with a competitive narrative which describes the news as fake in the first place.

Twitter data in conjunction with epidemiological models have already been used to study the spread of rumors and fake news by several authors [10, 16, 17, 26], where SIS and SEIZ compartmental models were employed to fit the data of the evolution of different news. Mounting experimental evidence highlights the strong link between digital media literacy and possibility to reliably identify the quality of online information. This connection has been early identified by communication scientists [20] and later confirmed by experimental studies, see e.g. [22, 24].

In [19], starting from an agent-based model for the dissemination of fake news in presence of competence, using the tools of kinetic theory, in the limit of a large number of agents, novel mathematical models were proposed and discussed. Previously, kinetic models that include the role of competence or knowledge had been proposed in [5, 29, 31]. The behavior of a social system composed by a large number of interacting agents has been studied in the case of opinion formation [3, 9, 14, 15, 34] and more recently epidemiological dynamics [1, 2, 12]. We refer to [28] for an introduction to the subject.

The compartmental structure of the model for fake-news spreading in presence of competence introduced in [19] is composed by four groups of individuals: the susceptible (S) agents—defined as the ones who are unaware of the fake news; the exposed (E) agents—those who know the news but still have not decide whether to spread it or not; the infectious (I) agents—who actively divulge and finally the skeptical or removed (R) agents—those who are aware of the news but choose to not spread it. On a population divided among such categories, there is also a social structure based on an additional time evolving variable that measures the *competence* level of the agents. Although the model has shown the capacity to correctly describe the role of competence in the dynamics of fake-news, its mathematical structure based on kinetic partial differential equations is generally too complex to be interfaced with the available data.

In an attempt to address this problem, in the present work by exploiting the knowledge of the equilibrium states of the corresponding mean-field model we derived reduced order macroscopic models based on ordinary differential equations in which, however, the role of competence continues to be present. The new social models, thanks to their simpler structure, are more suitable for data-driven applications. We emphasize that the methodology here adopted is quite general and that in principle points the way to introducing additional social characteristics of individuals into tractable mathematical models in terms of structural complexity.

The rest of the manuscript is organized according to the following sections. In Sect. 2, we recall the basic concepts of the kinetic model for describing the spread of fake-news in the presence of competence. Next, in Sect. 3, using the local equilibrium states of the competence we derive reduced order models that depend on the specific shape of the interaction function. Section 4 is devoted to presenting a series of numerical experiments in which we first validate the model and then consider data-driven applications based on Twitter. In the last section, a series of final considerations are reported.

## Kinetic models, competence and fake news spreading

In this section we present a model for the description of the spreading of fake news in a society characterized by a heterogeneous competence of agents. Our starting point is the compartmental kinetic approach recently proposed in [19]. We suppose that the system of agents can be divided in the following epidemiologically relevant states: susceptible (S) agents are the ones that are unaware of fake news, we further denote as exposed (E) the agents that encountered the fake news but have still to spread them, infectious (I) agents are the real spreader and, finally, the removed (R) agents are not actively engaged in the spread of misinformation. In the following we indicate with $\mathcal {C}=\{S,E,I,R\}$ the set of epidemiological compartments.

Aiming to incorporate the effects of personal competence on the fake news dynamics, we stick to a simple mathematical setting where the state of the individuals in each compartment, at any time $t\ge 0$, is characterized by the sole competence level $x\in {\mathbb {R}}_+$. Hence, we denote by $f_S = f_S(x, t)$, $f_E = f_E(x,t)$, $f_I = f_I(x,t)$, and $f_R = f_R(x,t)$ the distribution of competence at time $t \ge 0$ of susceptible, exposed, infectious and removed individuals, respectively. We neglect natality and mortality dynamics since we can consider a short time dynamic where nobody enters or leaves it during the spreading of the fake news. This assumption can be justified based on the average lifespan of fake news. Therefore, we can fix the total distribution of competence of a society to be a probability density for all $t \ge 0$

$$\begin{aligned} \int _{{\mathbb {R}}_+}\left( f_S(x,t) + f_E(x,t) + f_I(x,t) + f_R(x,t) \right) \, \mathrm dx = 1,\quad t > 0, \end{aligned}$$

Consequently, the quantities

$$\begin{aligned} S(t)&= \int _{{\mathbb {R}}_+} f_S(x,t)\, \mathrm dx,&E(t)&= \int _{{\mathbb {R}}_+} f_E(x,t)\, \mathrm dx,\\ I(t)&= \int _{{\mathbb {R}}_+} f_I(x,t)\, \mathrm dx,&R(t)&= \int _{{\mathbb {R}}_+} f_R(x,t)\, \mathrm dx \end{aligned}$$

denote the fractions of the population that are susceptible, exposed, infected, or recovered respectively at time $t\ge 0$. We also denote with $m_J^p(t)$ the moment of the distribution $f_J(x,t)$, $J \in {\mathcal {C}}$, of order $p\ge 0$

$$\begin{aligned} m^p_S(t)&= \frac{1}{S(t)}\int _{{\mathbb {R}}_+} x^p f_S(x,t)\, \mathrm dx,&m^p_E(t)&= \frac{1}{E(t)}\int _{{\mathbb {R}}_+} x^p f_E(x,t)\, \mathrm dx, \\ m^p_I(t)&= \frac{1}{I(t)}\int _{{\mathbb {R}}_+} x^p f_I(x,t)\, \mathrm dx,&m^p_R(t)&= \frac{1}{R(t)}\int _{{\mathbb {R}}_+} x^p f_R(x,t)\, \mathrm dx. \end{aligned}$$

Unambiguously we will indicate with $m_J(t)$, $J\in {\mathcal {C}}$, the mean values corresponding to $p = 1$.

### Competence and learning in multi-agent systems

Drawing inspiration from seminal models for multi-agent systems in presence of personal competence [29, 31] we introduce a binary interaction term expressing two different processes:

(i) learning processes by less competent agents that can learn from the more competent ones
(ii) the competence evolution depends by a social background in which individuals grow.

The dynamics described at point (i) can be easily sketched by the following process: if two agents belonging to compartment $H, J \in {\mathcal {C}}$ and characterized by competence levels $x,x_* \in {\mathbb {R}}_+$ meet, their post-interaction competence is given by1where $\lambda _H(\cdot )$, $H \in {\mathcal {C}}$, quantify the amount of competence lost by individuals of compartment *H* by the natural process of forgetfulness and the parameter $\lambda _{CH}$, $H \in {\mathcal {C}}$, models the competence gained through the interaction with members of the class *J*, with $J \in {\mathcal {C}}$. A possible choice for $\lambda _{CJ}(x)$ is $\lambda _{CJ}(x) = \lambda _{CJ}\chi (x \ge {\bar{x}})$, where $\chi (\cdot )$ is the characteristic function and ${\bar{x}} \in X$ a minimum level of competence required to the agents for increasing their own skills by interactions. In (1) $\eta _{HJ}$ and $\eta _{JH}$ are centered iid random variable such that, denoting by $\left\langle \cdot \right\rangle$ their expectation, we have $\left\langle \eta _{HJ}^2 \right\rangle = \left\langle \eta _{JH}^2 \right\rangle = \sigma ^2_{HJ}$.

We suppose that the process defined in (ii) takes place in a different time scale from the one of interactions between agents. In particular, unlike [19] we assume that the time scale of online interactions for competence formation is faster than interactions with the social background. To this end, we will consider advection terms that will be defined in the next section.

#### Remark 1

It is reasonable to assume that both the processes of gain and loss of competence from the interaction with other agents in (1) are bounded by zero. Therefore we suppose that if $J, H \in \lbrace {S, E, I, R}\rbrace$, and if $\lambda _J \in [\lambda _J^-, \lambda _J^+]$, with $\lambda _J^- > 0$ and $\lambda _J^+ < 1$, and $\lambda _{CJ}(x) \in [0, 1]$ then $\eta _{HJ}$ may, for example, be uniformly distributed in $[-1 + \lambda _J^+, 1 - \lambda _J^+]$.

### Fake news spreading in presence of a social feature

**Fig. 1:**
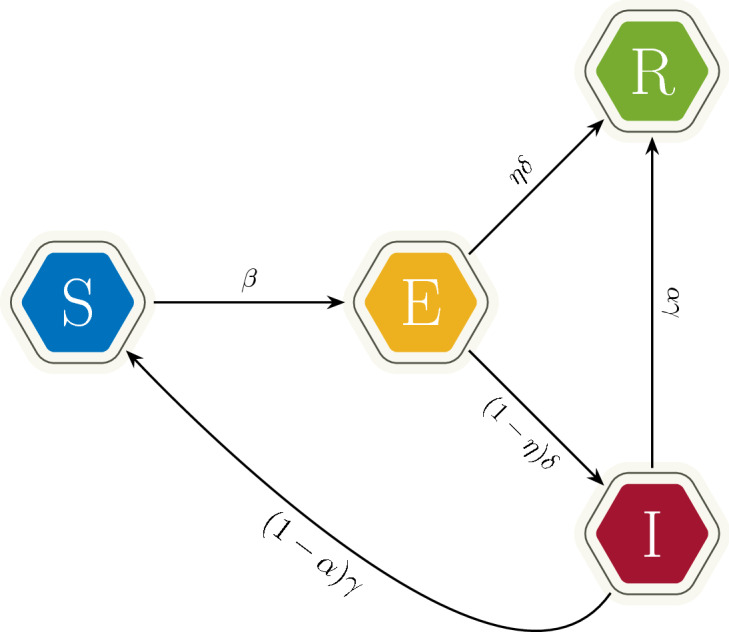
Dissemination dynamics for the SEIR model (2)

Following [19] we choose to describe the dissemination of fake news through a population of agents via a kinetic compartmental model. In this setting the description of the sole spreading dynamics can be illustrated by the following system of ODEs2Borrowing from the consolidated epidemiological tradition, we will refer to it as the SEIR model. System (2) describes the evolution of the mass fractions of the population that belongs to each compartment $J \in {\mathcal {C}}$ for each time $t \ge 0$. The parameters appearing in system (2) are presented in Table 1. Also a schematic representation of system (2) is given in Fig. 1. The last equation of system (2) translates the fact that—as specified at the beginning of the Section—the total mass of the population is preserved.

The combination of the learning mechanisms presented in the previous subsection together with the spreading of the fake news is described by the following kinetic model:3where the parameter $\epsilon$ describes the intensity of the interactions.

**Table 1:** Parameters definition in the SEIR model (3)

Parameter	Definition
$\beta$	Contact rate between susceptible and infected individuals
$1/\delta$	Average decision time on whether or not to spread fake news
$\eta$	Probability of deciding not to spread fake news
$1/\gamma$	Average duration of a fake news
$\alpha$	Probability of remembering fake news

In (3) the functional

4 $$\begin{aligned} K(f_S, f_I)(x,t) = f_S(x,t) \int _{{\mathbb {R}}^+} \kappa (x,x_*) f_I(x_*,t)\, \mathrm dx_*\end{aligned}$$

is the local incidence rate and $\kappa (x,x_*)$ is a nonnegative contact function measuring the impact of competence in the spreading of fake news. This function is decreasing with respect to the competences $x,x_*\ge 0$ of the population of susceptible and infected agents. In the following we will investigate the macroscopic effects of the following two choices of $\kappa (x,x_*)$

(A) Strong competence-based contact function $\kappa (x,x_*) = \beta /(x\,x_*)$, with $\beta >0$,
(B) Weak competence-based contact function $\kappa (x,x_*) = \beta e^{-x-x_*}$, $\beta >0$.

The two functions are both decreasing but have strong differences for $x,x_*\ll 1$. Indeed, since (A) is not limited for small competences it enforces the spreading of fake news among less competent agents compared with (*B*). Indeed, the function in (B) is bounded in ${\mathbb {R}}_+$. We further remark that individuals have the highest rates of contact with people belonging to the same social class, and thus with a similar level of competence.

#### Remark 2

In (3) the parameters are considered to be dependent on the competence level *x*, in general. This is to reflect the fact that competence plays a role in the dissemination of fake news.

Furthermore, the operators ${\mathcal {Q}}_{HJ}(f_H,f_J)(x,t)$, $J\in \mathcal {C}$, describe the binary collisions (1) and they determine the thermalization of the distribution of competence characterizing the *J*th compartment. The advection terms in (3) come models the influence of the social background on the competence dynamics. It is worth to observe that the evolution of mass fractions *J*(*t*) obeys the classical SEIR model with reinfection (2) by choosing ${\mathcal {Q}}_{HJ}\equiv 0$ and $\kappa (x,x_*) = \beta >0$. This would correspond in considering the spreading a fake news independent of the competence level of a system of agents.

In more details, we will consider the operators ${\mathcal {Q}}_{HJ}$ as integral operators that modify the competence distribution through repeated interactions of type (1) among individuals. We can fruitfully define the introduced operators in weak form as follows

5 $$\begin{aligned} \int _{{\mathbb {R}}^+}\varphi (x) {\mathcal {Q}}_{HJ}(f_H,f_J) \, \mathrm dx = \left\langle { \int _{{\mathbb {R}}^2_+} \bigl (\varphi (x') - \varphi (x)\bigr )\,f_H(x,t) f_J(x_*,t) \, \mathrm dx_*\mathrm dx}\right\rangle , \end{aligned}$$

where $\varphi (\cdot )$ is a test function and where the brackets $\left\langle {\cdot }\right\rangle$ indicate the expectation with respect to the random variables $\eta _{HJ},\tilde{\eta }_{HJ}$.

In the model (3) the function $\gamma (x)>0$ determines the duration of the fake news and can be strongly influenced by the competence level of the spreader. Furthermore, the function $\delta (x)>0$ is related to the average time that an agent eventually spend before the diffusion of a fake news such that people with high competence invest more time in checking information reliability, and $\eta (x) \in [0,1]$ characterizes individuals’ decision to spread fake news. The function $\alpha (x) \in [0,1]$ describes the probability to remember fake news and can be thought less influenced by the competence variable. In Table 1 we summarize all the introduced parameters.

### Asymptotic states of the learning process

We focus now on the learning dynamics introduced in model (3) whose evolution is given by the nonlinear operators ${\mathcal {Q}}_{HJ}(f_H,f_J)$, $H,J \in {\mathcal {C}}$, defined in (5). We concentrate in particular on the analysis of asymptotic states of the learning dynamics undergoing elementary interactions (1). We are therefore interested in the asymptotic distribution of the Boltzmann-type model

6 $$\begin{aligned} \begin{aligned} \dfrac{d}{dt} \int _{{\mathbb {R}}_+} \varphi (x) f_H(x,t)dx&=\sum _{J \in {\mathcal {C}}} \int _{{\mathbb {R}}^2_+} \langle \varphi (x') - \varphi (x)\rangle \,f_H(x,t) f_J(x_*,t) \, \mathrm dx_*\mathrm dx \\&= \dfrac{1}{2} \sum _{J \in {\mathcal {C}}} \int _{{\mathbb {R}}^2_+} \langle \varphi (x') + \varphi (x_*^\prime )- \varphi (x) -\varphi (x_*)\rangle \,f_H(x,t) f_J(x_*,t) \, \mathrm dx_*\mathrm dx. \\ \end{aligned} \end{aligned}$$

It is easily observed that if $\varphi (x) = 1$ the mass is conserved in (6) corresponding to the conservation of the total number of agents. If $\varphi (x) = x$ in (6) we obtain the evolution of the average competence in each compartment that is not conserved in time

$$\begin{aligned} \begin{aligned} \dfrac{d}{dt} (H(t)m_{H}(t))&= \sum _{J \in {\mathcal {C}}} \int _{{\mathbb {R}}_+^2} \left\langle x^\prime -x\right\rangle f_H(x,t)f_J(x_*,t)\mathrm dx\,\mathrm dx_* \\&=H(t) \sum _{J \in {\mathcal {C}}} J(t)(\lambda _{CJ}m_J(t) - \lambda _H m_H(t)), \end{aligned} \end{aligned}$$

and the total competence is conserved

$$\begin{aligned} \dfrac{d}{dt} \sum _{H \in {\mathcal {C}}} \int _{{\mathbb {R}}_+} x f_H(x,t)\mathrm dx = 0. \end{aligned}$$

Since the steady state solution of (6) is difficult to obtain, we can formally derive a simplified Fokker–Planck model in which the study of the asymptotic properties is much easier. To this end, we introduce the following quasi-invariant scaling of the relevant parameter of the binary scheme (1) given by

7 $$\begin{aligned} \lambda _H \rightarrow \tau \lambda _H, \qquad \lambda _{CH} \rightarrow \tau \lambda _{CH}, \qquad \sigma _{HJ}^2 \rightarrow \tau \sigma _{HJ}^2, \end{aligned}$$

with $\tau >0$. It is worth to mention that the introduced scaling is inspired by the so-called grazing collision limit of the Boltzmann equation, see [6, 36]. In the context of multi-agent systems this scaling has been introduced in [8, 33].

In the introduced regime of parameters the interactions become quasi-invariant, in the sense that the post-interaction competences $(x^\prime ,x_*^\prime )$ are such that $x^\prime -x$ and $x_*^\prime -x_*$ are small for $\tau \ll 1$. Hence, assuming $\varphi \in {\mathcal {C}}_0$, we can perform the following Taylor expansion

$$\begin{aligned} \begin{aligned} \varphi (x^\prime )-\varphi (x)&= (x^\prime -x)\dfrac{\mathrm d}{\mathrm dx}\varphi (x) + \dfrac{1}{2} (x^\prime -x)^2 \dfrac{\mathrm d^2}{\mathrm dx^2}\varphi (x)+ \dfrac{1}{6} (x^\prime -x)^3\dfrac{\mathrm d^3}{\mathrm dx^2}\varphi ({\bar{x}}), \end{aligned} \end{aligned}$$

with ${\bar{x}} \in (\min \{x,x^\prime \},\max \{x,x^\prime \})$. Hence, in the time scale $t/\tau$ we have

$$\begin{aligned}&\frac{\mathrm d}{\mathrm dt} \int _{{\mathbb {R}}_+} \varphi (x) f_H(x,t)\mathrm dx \\&\quad = \sum _{J \in {\mathcal {C}}} \int _{{\mathbb {R}}^2_+} (-\lambda _H(x)x + \lambda _{CJ}(x)x_*)\frac{\mathrm d\varphi (x)}{\mathrm dx}f_H(x,t)f_J(x_*,t)\,\mathrm dx_*\,\mathrm dx \\&\quad \quad + \sum _{J \in {\mathcal {C}}} \frac{\sigma _{HJ}^2}{2} \int _{{\mathbb {R}}_+^2} x^2 \frac{\mathrm d^2\varphi (x)}{\mathrm dx^2} f_H(x,t)f_J(x_*,t)\,\mathrm dx_*\,\mathrm dx + \sum _{J \in {\mathcal {C}}} R_{\varphi }(f_H,f_J), \end{aligned}$$

where we exploited the fact that $\left\langle \eta _{HJ} \right\rangle = 0$ and we have defined the sum of reminder terms

$$\begin{aligned} \begin{aligned} R_{\tau }(f_H,f_J)&= \frac{1}{2} \int _{{\mathbb {R}}_+^2} \tau (-\lambda _H(x)x + \lambda _{CJ}(x)x_*)^2 \frac{\mathrm d^2\varphi (x)}{\mathrm dx^2} f_H(x,t)f_J(x_*,t)\mathrm dx \,\mathrm dx_* \\&\quad + \frac{1}{6} \int _{{\mathbb {R}}_+^2} \frac{\left\langle (-\lambda _H(x)x + \lambda _{CJ}(x)x_* + \eta _{HJ}x)^3 \right\rangle }{\tau } \frac{\mathrm d^3\varphi (x)}{\mathrm dx^3} f_H(x,t) f_J(x_*,t)\mathrm dx\,\mathrm dx_* \end{aligned} \end{aligned}$$

We may observe that, assuming $\left\langle |\eta _{HJ}|^3\right\rangle <+\infty$, then we may write $\eta _{HJ} = \sqrt{\sigma ^2}\tilde{\eta }_{HJ}$, where we introduced the centered random variable $\tilde{\eta }_{HJ}$ with unitary variance and such that $\left\langle |\tilde{\eta }|^3 \right\rangle <+\infty$. Therefore, $\left\langle |\eta _{HJ}|^3 \right\rangle = (\sigma ^2)^{3/2} \left\langle |\tilde{\eta }_{HJ}|^3 \right\rangle$ and, under the scaling (7), we get $\left\langle |\eta _{HJ}|^3 \right\rangle \sim \tau ^{3/2}\sigma ^{3/2}$ . Hence, under the above assumption, proceeding as in [8] we can prove that for $\tau \rightarrow 0^+$

$$\begin{aligned} |R_\varphi (f_H,f_J)(x,t)| \rightarrow 0. \end{aligned}$$

Therefore in the new time scale, for $\tau \rightarrow 0^+$ and under the quasi-invariant scaling (7), we can show that the solution of model (6) converges to

8 $$\begin{aligned}&\frac{\mathrm d}{\mathrm dt} \int _{{\mathbb {R}}_+} \varphi (x) f_H(x,t)\mathrm dx \nonumber \\&\quad = \sum _{J \in {\mathcal {C}}} \int _{{\mathbb {R}}^2_+} (-\lambda _H(x)x + \lambda _{CJ}(x)x_*)\frac{\mathrm d\varphi (x)}{\mathrm dx}f_H(x,t)f_J(x_*,t)\,\mathrm dx_*\,\mathrm dx\nonumber \\&\quad \quad + \sum _{J \in {\mathcal {C}}} \dfrac{\sigma _{HJ}^2}{2} \int _{{\mathbb {R}}_+^2} x^2 \frac{\mathrm d^2\varphi (x)}{\mathrm dx^2} f_H(x,t)f_J(x_*,t)\,\mathrm dx_*\,\mathrm dx. \end{aligned}$$

Integrating back by parts we have obtained

9 $$\begin{aligned} \partial _t f_H(x,t) = \partial _x \left[ ( \lambda _H(x)x - \sum _{J \in {\mathcal {C}}} \lambda _{CJ}(x)J(t)m_J(t)) f_H(x,t) + \dfrac{\sigma ^2}{2}\partial _x(x^2 f_H(x,t)) \right] , \end{aligned}$$

with $\sum _{J \in {\mathcal {C}}} \sigma _{HJ}^2 = \sigma ^2$, coupled with the following boundary conditions

$$\begin{aligned} ( \lambda _H(x)x - \sum _{J \in {\mathcal {C}}} \lambda _{CJ}(x)J(t)m_J(t)) f_H(x,t) + \dfrac{\sigma ^2}{2}\partial _x(x^2 f_H(x,t)) \Big |_{x = 0} = 0, \end{aligned}$$

and

$$\begin{aligned} \dfrac{\sigma ^2}{2}\partial _x(x^2 f_H(x,t)) \Big |_{x=0} = 0, \end{aligned}$$

for all $H \in {\mathcal {C}}$. Assuming then $\lambda _{CJ} = \lambda _H = \lambda$ independent by $x \in {\mathbb {R}}_+$ for all $J,H \in {\mathcal {C}}$ the steady states $f_H^\infty (x)$, $H \in {\mathcal {C}}$ are solution of

$$\begin{aligned} \lambda ( x - m) f_H^\infty (x) + \dfrac{\sigma ^2}{2}\partial _x(x^2 f_H^\infty (x)) = 0, \end{aligned}$$

where $m = \sum _{J \in {\mathcal {C}}} J(t)m_J(t)$ is a conserved quantity as we already observed. Hence, we obtain that the large time distribution is an inverse Gamma

$$\begin{aligned} f_H^\infty (x) = H \dfrac{k^\mu }{\Gamma (\mu )} \dfrac{e^{-k/x}}{x^{1+\mu }}, \end{aligned}$$

where

$$\begin{aligned} \mu = 1+\dfrac{2\lambda }{\sigma ^2}, \qquad k = (\mu -1)m. \end{aligned}$$

Now, we highlight that for $t \rightarrow +\infty$ we have $f_E(x,t), f_I(x,t) \rightarrow 0$, which means that

$$\begin{aligned} f^\infty (x) = f_S^\infty (x) + f_R^\infty (x). \end{aligned}$$

In view of $S^\infty + R^\infty = 1$ we conclude that under the introduced assumptions

$$\begin{aligned} f_S^\infty (x) = S^\infty f^\infty , \qquad f_R^\infty = (1-S^\infty )f^\infty . \end{aligned}$$

## Reduced order models for fake news spread with competence

Once we have characterized the equilibrium distribution of the transition operators ${\mathcal {Q}}_{HJ}(\cdot ,\cdot )$, with $H,J \in \mathcal {C}$, we can study the complete system (3). The aim of this section is the definition of observable macroscopic equations of the introduced kinetic model.

Integrating both sides of (3) with respect to $x \in {\mathbb {R}}_+$ and recalling that the introduced operators are mass and momentum preserving, we obtain the following system for the evolution of the mass fractions *J*(*t*), $J \in {\mathcal {C}}$10whereas for the momentum we get11We can observe that the obtained system is not closed since the evolution of mass fractions *J*(*t*) and of the momentum depend on the evolution of the distribution functions $f_J(x,t)$. The closure of the obtained system can be obtained by formally resorting to a limit procedure. Indeed, assuming that the time scale involved in the process of competence formation is $\epsilon \ll 1$, we have a fast learning process of the system of agents with respect to the evolution of the spreading of fake news. Therefore, for $\epsilon \ll 1$ the distribution function $f_J(x,t)$ reaches fast the inverse Gamma equilibrium with mass fractions *J*(*t*) and local mean values $m_J(t)$.

In the following we obtain two different set of macroscopic equations in relation with the considered contact rate function $\kappa (x,x_*)$.

### Social closure with a strong competence-based contact function

We consider the case (*A*) introduced in Sect. 2.2 corresponding to a strong competence-based contact function defined by $\kappa (x,x_*) = \frac{\beta }{x x_*}$, $\beta >0$. We have12where

13 $$\begin{aligned} H_J(t) = \int _{{\mathbb {R}}^+} \frac{1}{x} f_J(x,t) \, \mathrm dx. \end{aligned}$$

Therefore, in the limit $\epsilon \rightarrow 0^+$ we can plug $f_J^\infty (x)$ in (13) which becomes

14 $$\begin{aligned} H_J(t) = \frac{\mu }{\mu - 1}\frac{1}{m_J(t)}, \end{aligned}$$

thanks to the properties of the inverse Gamma distribution, leading to15Next, looking at (11), recalling that under the hypothesis that $\lambda _J = \lambda _{CJ}$ for $J \in {\mathcal {C}}$, the knowledge exchange operator also preserves momentum, we have the following system of equations16which, using the fact that

$$\begin{aligned} \frac{\mathrm d[m_J(t)J(t)]}{\mathrm dt} = J(t) \frac{\mathrm dm_J(t)}{\mathrm dt} + m_J(t) \frac{\mathrm dJ(t)}{\mathrm dt}, \end{aligned}$$

implies17that is, we obtained a closed system of eight ordinary differential equations (15), (17).

### Social closure with a weak competence-based contact function

If, instead, we consider the case *B)* of Sect. 2.2, corresponding to the weak competence-based contact function defined by $\kappa (x,y) = \mathrm e^{-x}\mathrm e^{-y}$, it is possible to write

18 $$\begin{aligned} {\tilde{H}}_J(t) = \int _{{\mathbb {R}}_+} e^{-x}f_J(x,t)dx. \end{aligned}$$

As discussed in Sect. 3.1, in the limit $\epsilon \rightarrow 0^+$ we may plug the asymptotic distribution $f_J^\infty$ of the Fokker–Planck model (9) in (18) to obtain

$$\begin{aligned} \begin{aligned} {\tilde{H}}_J(t) = \underbrace{\frac{2(\mu -1)^{\mu /2} (m_J(t))^{\mu /2}\,{\mathbb {K}}_\mu (2\sqrt{(\mu -1)m_J(t)})}{\Gamma (\mu )}}_{{:}{=} C_\mu (m_J)}, \end{aligned} \end{aligned}$$

where ${\mathbb {K}}_a(x)$ stands for the modified Bessel function of the second kind of order *a* evaluated at *x*. Hence, if we consider system (10) under the assumption of weak competence-based contact function we obtain19which becomes20The next equation will help to close the system

21 $$\begin{aligned} \int _{{\mathbb {R}}^+} x\,f_J^\infty (t)\mathrm e^{-k/x}\mathrm e^{-x}\, \mathrm dx = m_J(t) J(t) C_{\mu - 1}(m_J), \end{aligned}$$

which is a straightforward consequence of the following property of the modified Bessel functions of the second kind

$$\begin{aligned} x\, {\mathbb {K}}_{\mu +1}(x) - 2\mu {\mathbb {K}}_\mu (x) = x\, {\mathbb {K}}_{\mu -1}(x), \quad x\in {\mathbb {R}}^+. \end{aligned}$$

Again under the assumptions that $\lambda _J = \lambda _{CJ}=\lambda$ for $J \in {\mathcal {C}}$, integrating with respect to *x* Eq. (11), with the aid of Eq. (21), we get22which, using again the fact that

$$\begin{aligned} \frac{\mathrm d[m_J(t)J(t)]}{\mathrm dt} = J(t) \frac{\mathrm dm_J(t)}{\mathrm dt} + m_J(t) \frac{\mathrm dJ(t)}{\mathrm dt}, \end{aligned}$$

leads to23

## Examples and applications

In this section we numerically validate the modeling framework proposed in (3) with local incidence rate 4 in the settings (A)–(B). We stress that those form of contact functions generate different macroscopic models that have been defined in (15), (17) and (20), (23), respectively, for $\epsilon \ll 1$. Once established the consistency of the approach, we proceed by exploiting the macroscopic sets of equations for calibration purposes based on a freely available repository for the spreading of hashtags linked to known fake news. The proposed data-oriented approach is fundamental to experimentally observe the different impact of the contact function in identifying impact of competence in the fake news dynamics.

From the numerical point of view we will exploit an implicit structure preserving method for the Fokker–Planck operator (9) based on the schemes presented in [32]. The advantage of these methods relies on an arbitrarily accurate description of the steady state distribution of the Fokker–Planck model of interest. Similar approaches have been investigated in a different context also in [12, 13, 30].

### Test 1: Validation of the social closure

In this first test we compare the evolution of mass fractions *J*(*t*) and means $m_J(t)$, $J \in {\mathcal {C}}$, obtained from direct integration of $f_J(x,t)$, solution to (3), with respect to the competence $x \in {\mathbb {R}}_+$, with the macroscopic models (15), (17) and (20)–(23) for several regimes of $\epsilon >0$. We start by outlining the procedure by which we solve the system of kinetic equations (3) with Fokker–Planck interaction operators. Since $\epsilon >0$ is assumed to be small, we adopt a time splitting procedure. In particular, upon introducing a time discretization $t^n = n\Delta t$, $\Delta t>0$ constant, we proceed as follows.

*I. Fokker–Planck solver.*    At time $t = t^n$, we determine the distributions $f_H(x,t)$ for all $H \in {\mathcal {C}}$ solution towhere ${\mathcal {Q}}(f_H)$ is the Fokker–Planck operator defined in Sect. 2.3 whose form, in the hypothesis $\lambda _H = \lambda _{CJ} = \lambda$, is given by

$$\begin{aligned} {\mathcal {Q}}(F_H)(x,t) =\partial _x \left[ \lambda (x-m)F_H + \dfrac{\sigma ^2}{2}\partial _x (x^2F_H(x,t)) \right] . \end{aligned}$$

**Fig. 2:**
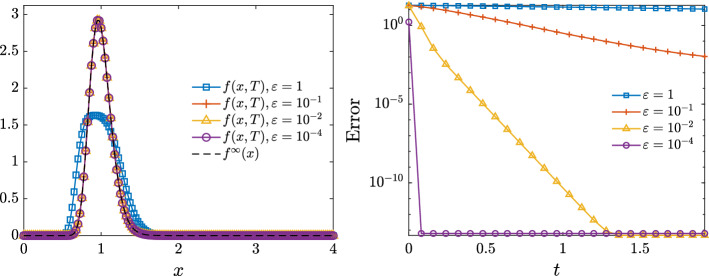
**Test 1**. Left: numerical distribution obtained with SP implicit scheme at time $T = 2$ for several $\epsilon = 1$, $10^{-1}$, $10^{-2}$, $10^{-4}$. Right: evolution of the $L^2$ error $\Vert f(x,T)-f^\infty (x) \Vert _{L^2}$. In all the tests we considered a discretization of the domain [0, 4] obtained with $N_v = 201$ grid points and $\Delta t = 10^{-4}$

In this step we take advantage of an implicit structure preserving (SP) scheme for Fokker–Planck equations [32] and describes with arbitrary accuracy the steady state of the model. In Fig. 2 we report for several $\epsilon = 1,10^{-1},10^{-2},10^{-4}$ the numerical solution of the Fokker–Planck model in the time interval [0, *T*], $T = 2$, obtained from a discretization of the domain [0, 4] with $N_v = 201$ grid points and with $\Delta t = 10^{-4}$. We may observe that the scheme is capable to approximate the inverse Gamma analytical equilibrium $f^\infty (x)$. We also report the evolution of the $L^2$ numerical error computed as $\Vert f(x,t)-f^\infty (x) \Vert _{L^2}$ in the time frame [0, 2] from which we can observe how for sufficiently small values of $\epsilon$ we correctly approximate the given equilibrium distribution.

*II. Advection-Reaction step.*   Hence, we consider the distribution obtained in the interaction step as an input for the advection-reaction dynamics for $t \in [t^{n+1/2},t^{n+1}]$In particular, we adopted a second order Lax-Wendroff scheme coupled with an explicit time integration.

In the test of this subsection, unless otherwise specified, we prescribe as initial datum the distribution

24 $$\begin{aligned} f_H(x,0) = H(t) \dfrac{a_1^{a_2}}{\Gamma (a_2)}x^{-a_2-1}\exp \{-a_1/x\}, \qquad H \in \{S,E,I,R\}, \end{aligned}$$

where $a_1 = 2(a_2-1)$ and $a_2 = 1.25$ with initial mass fractions

25 $$\begin{aligned} S(0) = 0.98, \qquad E(0) = 0.018, \qquad I(0) = R(0) = 0.001. \end{aligned}$$

**Fig. 3:**
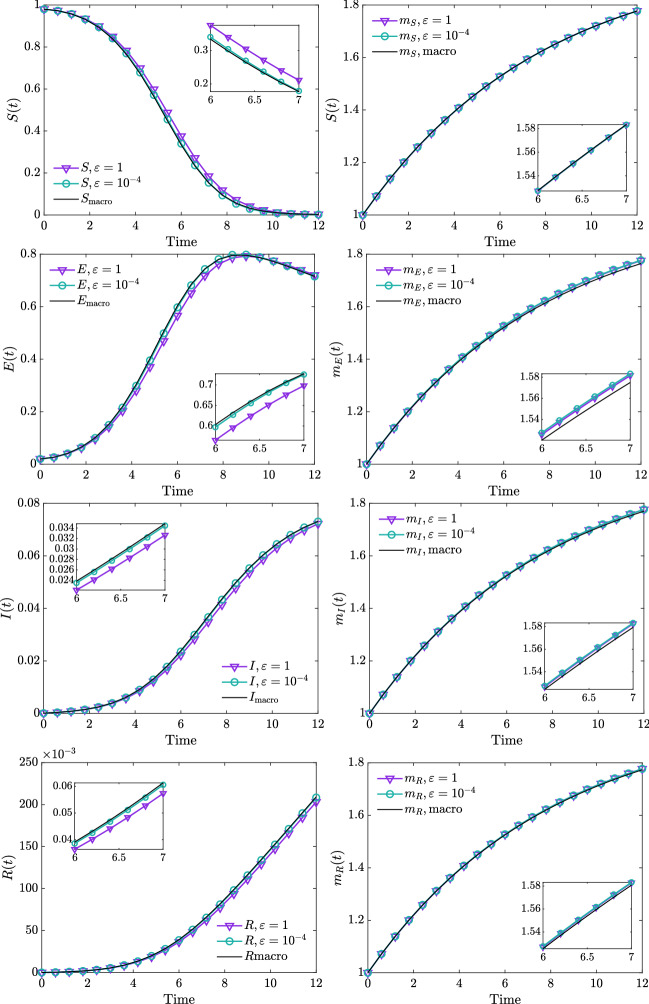
**Test 1**. Evolution of mass fractions (left) and mean values (right) obtained from direct integration of the kinetic model (3) in the case $\kappa (x,x_*) = \beta /(x\,x_*)$, for $\epsilon = 1$, $\epsilon = 10^{-4}$ together with the evolution of mass fractions of the macroscopic model (15)–(17). In both cases we considered $\alpha = 0.9$, $\beta = 20$, $\gamma = 0.2$, $\delta = 0.05$. The kinetic model has been solved through the scheme $\mathrm I$–$\mathrm {II}$ over the domain [0, 4], discretization obtained with $N_v = 201$ grid points and $\Delta t = 10^{-4}$. The initial distribution of the kinetic model has been defined in (24)–(25)

**Fig. 4:**
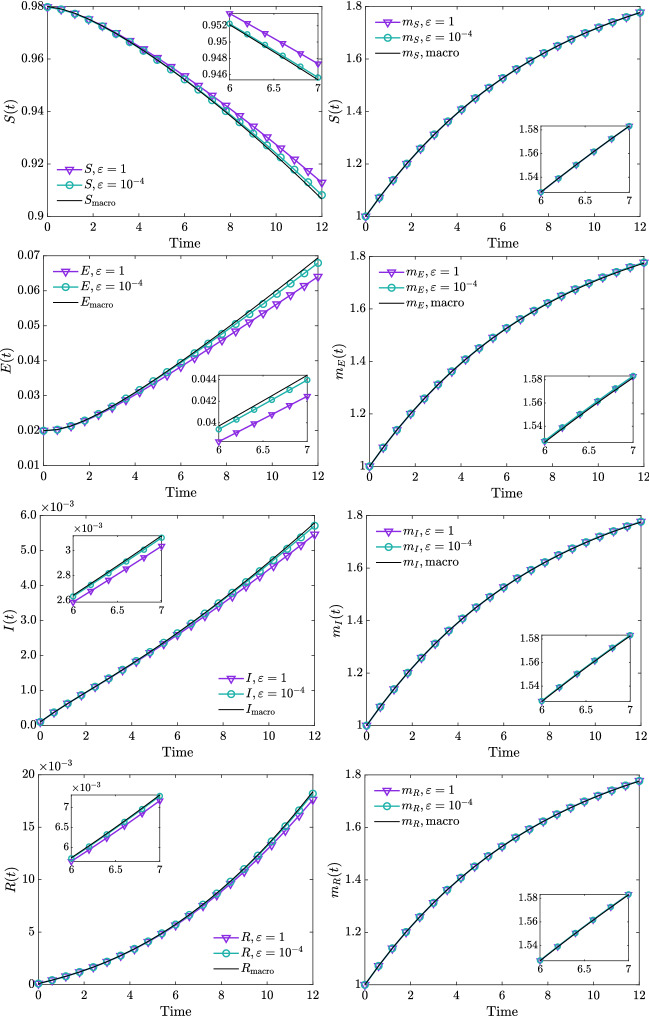
**Test 1**. Evolution of mass fractions obtained from direct integration of the kinetic model (3) in the case $\kappa (x,x_*) = \beta e^{-x-x_*}$, for $\epsilon = 1$, $\epsilon = 10^{-4}$ together with the evolution of mass fractions of the macroscopic model (15)–(17). In both cases we considered $\alpha = 0.9$, $\beta = 20$, $\gamma = 0.2$, $\delta = 0.05$. The kinetic model has been solved through the scheme I–II over the domain [0, 4], discretization obtained with $N_v = 201$ grid points and $\Delta t = 10^{-4}$. The initial distribution of the kinetic model has been defined in (24)–(25)

We consider the choice of parameters $m = \sum _{H \in \mathcal {C}}H(0)m_H(0)$, $\lambda = 0.25$ and $\sigma = 0.01$ for (4.1). The fake news dynamics is regulated by the following choice of parameters $\alpha = 0.9$, $\beta = 20$, $\gamma = 0.2$, and $\delta = 0.05$. For contact rates *A)*–*B)* we compared the evolution of mass fractions and mean values obtained from the integration of (3) with the ones derived in Sect. 3. We consider the time interval [0, *T*], $T = 12$, a uniform time discretization with $\Delta t = 10^{-4}$ and $\epsilon = 1$, $10^{-4}$. In particular, Fig. 3 refers to the case $\kappa (x,x_*) = \beta /xx_*$ and Fig. 4 to the case $\kappa (x,x_*)= \beta e^{-x-x_*}$. In both cases we may observe that for small values of $\epsilon$ the obtained macroscopic models are accurate in describing the trends of observable quantities of the kinetic field model. The macroscopic systems of coupled ODEs has been solved through a RK4 numerical scheme with $\Delta t = 10^{-4}$.

### Test 2: A data driven application to Twitter

In this test we focus on the spreading of the fake news by considering available Twitter data from the repository TweetSets.^1^ In details, we analyzed the evolution from March to November, 2020 of the hashtag #facemask related to the COVID-19 pandemic, and of the hashtags #hurricaneflorence#fakenews both associated to the hurricane Florence of September 2018 that caused catastrophic damages in USA, particularly in the states of North Carolina and South Carolina.

**Fig. 5:**
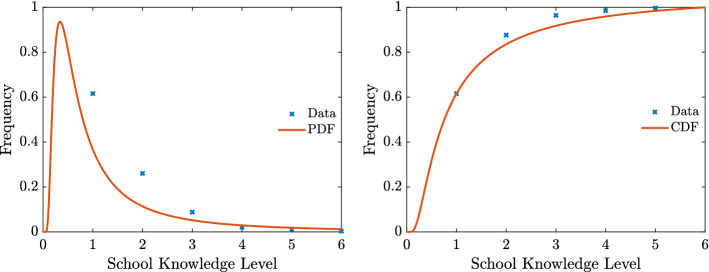
**Test 2**. Competence distribution and its inverse Gamma approximation *f*(*x*) (26) corresponding to $c_1 \approx 0.75$, $c_2 \approx 1.25$ and leading to a mean competence background of $m_B = 3$. Data refers to 2011 Italian census and are used as representative of a prototypical Western country. On the *x*-axis we indicated with (1) lower secondary education, (2) upper secondary education, (3) undergraduate, (4) master, (5) second level master, (6) doctorate

In the following we will assume that the competence variable is strongly related to the education level of a country. The data for the initial distribution of education has been extrapolated by the available Italian data from 2011 ISTAT census, and has been considered as representative data of a prototypical Western country [21]. As underlined in [21] the cumulative distribution of education exhibits a power-law type of tail. For this reason, as an approximation of the competence distribution we considered an inverse Gamma of the form

26 $$\begin{aligned} g(x) = \dfrac{c_1^{c_2}}{\Gamma (c_2)}\dfrac{e^{-c_1/x}}{x^{1+c_2}}, \end{aligned}$$

with $c_1,c_2>0$ obtained by data fitting. More precisely, we measure the education level on the scale [0, 6] where 6 represents the education of people with a PhD (see Fig. 5).

#### Test 2A: Fitting the model to data

Once we have obtained the initial competence distribution together with the value of $m_B$ we can estimate the parameters of the models defined in (15)–(17) and (20)–(23). Several approaches have been proposed in the literature, see e.g., [25]. It is worth to mention that several uncertainties are present in data linked to news-monitoring. For example the total population size is generally unknown and the total number of Twitter accounts represent an upper bound over the real active users.

The approach adopted in [16], and subsequently in [17, 26], is to treat this quantity as a parameter to be determined in the minimization process along with the parameters of the models. To reduce the number of parameters to optimize we follow a different path. In particular, as initial guess on the total population size, since the datasets that we used for the fitting were based on U.S. hashtags, we considered that each fake-news spreader has in average 453 followers.^2^ Hence, in average we may expect that the total number of susceptible is given by the total number of tweets multiplied by the average number of followers. To take also into account both the number of bots on Twitter as found in [35] (and references therein) and users whose activity could be not assiduous enough to matter during the lifespan of the considered fake news, the initial guess was also reduced by a factor of 4.

Let us denote by ${\hat{I}}(t)$ the number of active spreaders obtained from the data, while *I*(*t*) is the number of infectious agents given by the macroscopic differential model. Hence, we consider the following cost functional

$$\begin{aligned} F({\hat{I}}, I) = \bigg \Vert { \int _{t_0}^{t_f} {\hat{I}}(t)dt - \int _{t_0}^{t_f} I(t)dt} \bigg \Vert _{L^2([t_0,t_f])}, \end{aligned}$$

where $[t_0,t_f]$ is the time-frame (in h) during which we solve the minimization problem

27 $$\begin{aligned} \min _{\alpha ,\beta ,\gamma ,\delta \in {\mathbb {R}}_+} F({\hat{I}}, I), \end{aligned}$$

whereas $\eta$ was kept fixed and equal to 0.5.

Since data for the evolution of compartments *S*, *E*, *R* are not at our disposal, as well is not the initial means value for any of the compartments, we solved the ODE model on $[t_\star , t_0]$, where $t_0$ is the starting point of the spreading process and $t_\star$ is a suitable unknown time previous to $t_0$ starting from single exposed, infectious and recovered individuals. The idea is to simulate an initial situation for the spread of fake news to happen. Furthermore, we considered initial mean values equal to the half of the mean background distribution of competence, i.e. $m_J(0) = 1.5$.

**Fig. 6:**
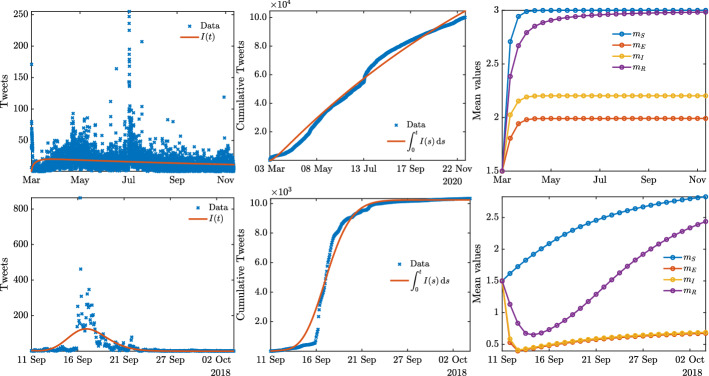
**Test 2A.** Top row: optimization results for #facemask. Bottom row: optimization results for #florence#fakenews. From right to left: approximation of raw data on the number of tweets, cumulative distribution and evolution of the mean competence for each compartment

In Fig. 6 we compare the evolution on the number of tweets regarding the hashtag #facemask, from 3rd March 2020 to 22 November 2020, and the hashtag #florence#fakenews, from 11th September 2018 to 4th October 2018, with the evolution of the model (15), (17). The obtained parameters are reported in Table 2.

In both cases, we may observe that the evolution of the mean competence levels are different in the four compartments and, in particular, that low competence levels are associated to exposed and infectious agents, i.e., the active spreaders. The outcome reflects the intuitive idea that the disinformation could be driven by the lack of capability to recognize an information as purposely false in the first place.

**Fig. 7:**
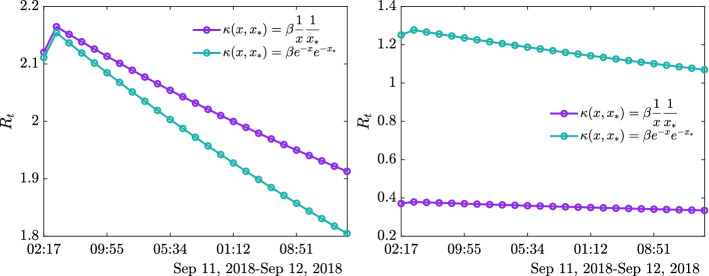
**Test 2A**. Evolution of $R_t$ in the first 24 h of datasets #facemask (left) and #florence#fakenews (right) for the parameters estimated in Table 2 relative to the introduced contact functions $\kappa (x,x_*)$

To better take into account the impact of a competence-based contact rate function $\kappa (x,x_*)$, we also computed the associated basic reproduction number $R_t$ using the parameters $\beta$ and $\gamma$ estimated previously for both datasets, reported in Table 2. Following [4, 19], and omitting the details for brevity, we consider a generalized version of the classical reproduction number defined as

28 $$\begin{aligned} R(t) = \frac{\int _{{\mathbb {R}}^+} K(f_S,f_I)(x,t)\,dx}{\int _{{\mathbb {R}}^+} \gamma f_I(x,t)\,dx}, \end{aligned}$$

where again we leveraged the structure preserving scheme proposed in [32] to perform the calculations (see Fig. 7).

**Table 2:** **Test 2A.** Estimated parameters for the entire datasets for the hashtags #facemask (second and third column) and #florence#fakenews (fourth and fifth column)

Parameter	#facemask		#florence#fakenews	
	$\kappa (x,x_*) = \beta /(x\, x_*)$	$\kappa (x,x_*)=\beta e^{-x-x_*}$	$\kappa (x,x_*) = \beta /(x\, x_*)$	$\kappa (x,x_*)=\beta e^{-x-x_*}$
$\alpha$	0.9995	0.9993	1.0000	0.9999
$\beta$	0.0122	0.2937	0.0901	0.9999
$\delta$	0.0237	0.0336	1.0000	0.1930
$\gamma$	0.0046	0.0079	0.2127	0.9999

#### Test 2B: Forecasting under data uncertainties

To analyze the impact of uncertainties in data and parameters we consider a 3D random variable ${\mathbf {z}} = (z_1,z_2,z_3)$ with distribution $\rho ({\mathbf {z}})$. We will suppose that the random vector ${\mathbf {z}}$ has independent components, i.e. $\rho ({\mathbf {z}}) = \rho _1(z_1)\rho _2(z_2)\rho _3(z_3)$. Taking into account parametric uncertainties, we consider the estimated model parameters as follows

29 $$\begin{aligned} \beta (z_1) = \beta _0 (1+ c_\beta z_1),\qquad \gamma (z_2) = \gamma _0 (1+ c_\gamma z_2), \qquad \delta (z_3) = \beta _0 (1+ c_\delta z_3), \end{aligned}$$

where we supposed $z_1,z_2,z_3 \sim {\mathcal {U}}([-1,1])$ and $c_\beta ,c_\gamma ,c_\delta >0$. As a result, the macroscopic quantities describing the evolution of compartments result affected by the introduced uncertainties increasing their dimensionality $J({\mathbf {z}},t)$, $m_J({\mathbf {z}},t)$, $J \in {\mathcal {C}}$. In order to handle efficiently the introduced uncertainties in the dynamics we adopt a stochastic collocation approach based on stochastic Galerkin methods, we refer the interested reader to [38] for an introduction and to [2, 39] for applications in compartmental modelling of epidemic dynamics. This class of methods allows to accurately quantify the propagation stochasticity in a parametric differential model when information on the uncertainties’ distribution are available. We remark that fast convergence properties hold under suitable regularity assumptions on the problem’s solution. In details, we construct a 3D sample $\{z_{i,k}\}_{k=0}^M$, $i=1,2,3$, obtained in a collocation setting through Gauss–Legendre polynomials with $M = 5$ nodes.

**Fig. 8:**
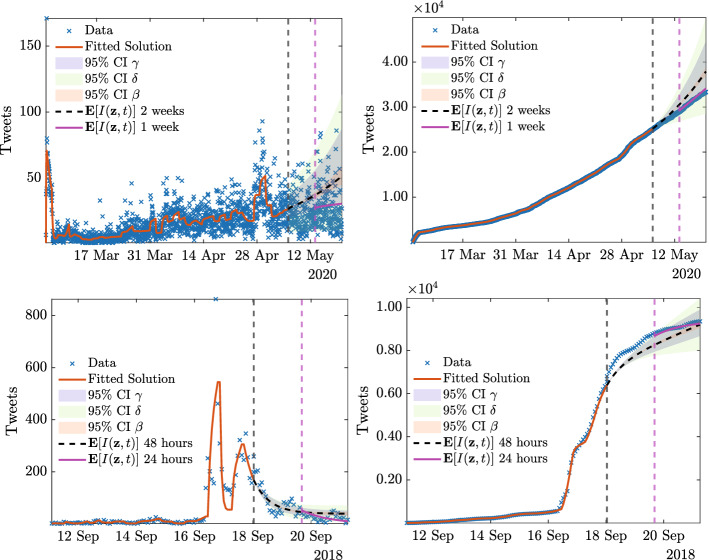
**Test 2B**. Top row: comparison between 14 days (May 3rd–May 14th, 2020) and 7 days (May 3rd–May 10th, 2020) predictions of #facemask based on the model (15), (17) with uncertain parameter (29) with $c_\beta = 200 \beta _0$, $c_\gamma = 2 \gamma _0$, $c_\delta = 2\times 10^4 \delta _0$. Bottom row: comparison between 24 h (September 18th–September 19th, 2018) and 48 h (September 18th–September 20th, 2018) predictions of #florence#fakenews based on the model (15), (17) with uncertain parameter (29) with $c_\beta = 0.2 \beta _0$, $c_\gamma = \gamma _0$, $c_\delta = 2\times 10^4 \delta _0$

In Fig. 8 we display the dynamics of the considered fake-news with respect to available data. In details, for #florence#fakenews we consider the period from September 11th 2018 to September 21st 2018. We consider two successive prediction horizons respectively of 1 day, i.e. the parameters of the models are calibrated taking into account data until September 19th, and a 2 days prediction horizon, where the calibration is based only on data until September 18th. Regarding #facemask we considered the period from March 3rd to May 17th. Also in this case we consider two successive prediction horizons of 1 week, i.e. the parameters of the models are calibrated taking into account data until May3rd, and a two weeks prediction horizon, where the calibration is based on data until May10th.

We highlight in dashed black and magenta the expected value of the predicted number of tweets ${\mathbb {E}}[I({\mathbf {z}},t)] = \int _0^1 I({\mathbf {z}},t)\rho _1(z_1)\rho _2(z_2)\rho _3(z_3)dz_1 dz_2 dz_3$. Together with the expected trends we plot the $95\%$ confidence intervals (CI) with respect to the random parameters $\beta (z_1)$, $\gamma (z_2)$, and $\delta (z_3)$. The blue shaded band is relative to the variability in $\gamma (z_2)$, the green shaded to the variability in $\delta (z_3)$ whereas the shaded red is relative to the variability in $\beta (z_1)$.

### Test 3: Competence background in misinformation

In this test we perform a retrospective analysis to study how the background could influence the dissemination of fake news as a result of a different learning process. We recall that the background modifies through a learning dynamic the effectiveness of the level of knowledge in identifying fake news. As a consequence high values of the background correspond to a high level of effectiveness of the competence while low values will make it difficult to identify the fake-news. Indirectly, the background acts as a control term which limits the spread of the misinformation. This can also be interpreted as a process of education specific to the identification of fake news that allows to limit the so-called knowledge neglect phenomenon [18].

We consider the two datasets for the hashtags #facemask and #florence#fakenews with the estimated parameters reported in Table 2 and we increase the value of the competence level attained by the background, i.e., $m_B$, while keeping fixed the parameters during the dynamics defined by (15), (17) and (20), (23).

**Fig. 9:**
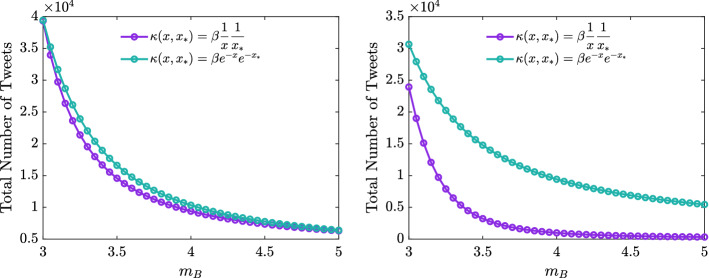
**Test 3.** Total number of infectious agents for the hashtags #facemask (left) and #florence#fakenews (right) as a function of the competence background. In both cases, we employed the parameters reported in Table 2

Hence, we performed the test with both choices of a strong and weak competence based contact function; the results are summarized in Fig. 9. In all cases, we see how increasing the competence of the background reduces the spread of fake news, leading to a decrease in the cumulative number of tweets of infectious agents proportional to the increase in the value of $m_B$. Indeed, we can observe how increasing the competence of the background, we obtain an evident decrease in the overall misinformation for both the examples considered #facemask and #florence#fakenews.

## Concluding remarks

Despite the digital transformation of governments and the modernization of public administration, a global decline in democracy is occurring around the world. The spread of fake news created for the purpose of polarizing society in certain directions poses a risk to democratic institutions. The role of individuals’ knowledge and the ability to use it in identifying false information is deemed of paramount importance.

In this paper starting from a model for the description of fake-news dissemination in the presence of heterogeneous agents with different levels of competence, through the tools of kinetic theory, reduced-order models have been derived that allow to keep the effects of the of competence in the dynamics and that, thanks to their simplified structure, can be interfaced with data.

The starting model is inspired, as in much of the literature related to fake-news, to the epidemiology, so it is based on a compartmental structure. The introduction of competence allows to analyze complex phenomena of great relevance in contemporary society, such as the effectiveness of control actions taken to limit the spread of fake news and the role of knowledge neglect in misinformation.

The methodology adopted in this article is fully general and depends closely on the equilibrium state of the social variable and the social interaction function at the basis of fake-news spreading. As a consequence, additional social variables that play a key role in the spread of misinformation may be embedded in the dynamics using similar arguments. The ability to have a model that can be interfaced with the available data allowed us to present some preliminary examples of applications to the case of fake-news spreading on Twitter.

## Data Availability

The datasets generated during the current study is available from the corresponding author on reasonable request. The datasets analysed during the current study are freely available from the website https://doi.org/10.5281/zenodo.1289426.

## Footnotes

1 Justin Littman. (2018). TweetSets. Zenodo. https://doi.org/10.5281/zenodo.1289426.

2 Kickfactory, link at https://kickfactory.com/blog/average-twitter-followers-updated-2016/, last accessed: 21th February 2022.
